# Physicians' Knowledge, Attitudes and Barriers Regarding Chronic Kidney Disease Diagnosis and Management in Saudi Arabia

**DOI:** 10.7759/cureus.50247

**Published:** 2023-12-09

**Authors:** Naweed Al-Zaman, Alaa Alem, Ohood A Alharbi, Ebtesam Ahmed Abdullah, Duha W Azouni, Raghad K Hammad, Reem M Alhejaily, Muayad Albadrani

**Affiliations:** 1 Internal Medicine, College of Medicine, Taibah University, Medina, SAU; 2 Medicine, College of Medicine, Taibah University, Medina, SAU; 3 Family and Community Medicine, College of Medicine, Taibah University, Medina, SAU

**Keywords:** physicians' barriers, physicians' knowledge, physicians' practice, internal medicine, family medicine, general practitioners, saudi arabia, chronic kidney disease

## Abstract

Background: Chronic kidney disease (CKD) is defined as abnormalities of kidney structure and/or function persisting for a minimum of three months.

Methods: An online cross-sectional study was conducted in the Kingdom of Saudi Arabia (KSA) between June and August 2022 to assess the knowledge, attitudes, practices, and barriers among family, internal medicine, and general physicians related to CKD screening, diagnosis, and management.

Results: A total of 427 physicians were included in the study. The majority exhibited a strong grasp of the accurate definition of CKD (83%) and recognized common risk factors, such as diabetes (99%), drugs (95%), and hypertension (98%). Two-thirds of physicians were aware of the five stages of CKD and identified estimated glomerular filtration rate (eGFR) and creatinine clearance as the most suitable markers for kidney function. Physicians also displayed knowledge of CKD-related complications. However, a noticeable gap between knowledge and practice was evident. Only one-third of participants reported screening their patients every year, primarily using serum creatinine (92.5%) and eGFR (97%) for diagnosis, while only 16% recognized that stage 4 CKD is the appropriate time to refer patients to nephrologists. In terms of barriers, the majority of physicians reported encountering low barriers to CKD management, but general practitioners working in primary healthcare centers experienced significantly higher levels of barriers.

Conclusion: Most participants in our study possess a good level of knowledge and positive attitudes towards CKD diagnosis and management. Nevertheless, a discrepancy between knowledge and practical application, particularly in terms of over-screening and early referral, highlights the need for educational efforts to improve physician practice in KSA. These findings underscore the importance of addressing this gap to promote effective CKD management.

## Introduction

The Kidney Disease Improving Global Outcome (KDIGO) defines chronic kidney disease (CKD) as abnormalities of kidney function or structure, present for more than three months, with health implications. KDIGO classification is based on the cause, with five stages determined by the estimated glomerular filtration rate (eGFR) category, and three stages determined by the albuminuria category [1]. CKD is a global health problem with a worldwide prevalence of CKD at 13.4% [2]. In the Kingdom of Saudi Arabia (KSA), the prevalence of CKD was found to be 5.7% based on a community-based pilot study. Additionally, the study revealed that individuals over the age of 60 (25%), those with hypertension (HTN) (58.33%), and those with diabetes mellitus (DM) (58.33%) had a higher CKD prevalence [3].

A full medical history, physical examination, and investigations are essential for CKD evaluation. Screening for CKD includes measurement of serum creatinine, eGFR using a serum creatinine-based equation, and measurement of the urine albumin to creatinine ratio (ACR). Urinalysis with microscopic urine sediments examination is helpful if intrinsic renal disease is suspected. Also, renal ultrasonography is recommended to evaluate for structural abnormalities [4].

CKD progression is associated with multiple complications, some of which are well-described and measurable, such as anemia, cardiovascular disease, HTN, volume overload, electrolyte imbalance, mineral bone disorder, and acid-base abnormalities. Other complications have less clear pathogenesis and are less well-defined, including anorexia, nausea, pruritus, fatigue, cachexia, and sexual dysfunction. These may manifest as complex symptoms often linked to advanced CKD [5].

Therapeutic interventions can effectively manage complications associated with CKD and slow down the progression of the disease. The management of CKD includes treatment of albuminuria, reducing cardiovascular risk, avoidance of nephrotoxic drugs, and adjusting doses based on renal function [6]. Early detection is crucial for slowing the progression of CKD to advanced stages, which is linked to adverse outcomes and high mortality. Referral to a nephrologist is essential for evaluating and planning for renal replacement therapy (RRT) [6]. Referral should be initiated when patients reach stage 4 CKD (eGFR less than 30 mL/min/1.73 m2) as per KDIGO guidelines [1].

Primary healthcare physicians in the United States of America (USA) identified multiple barriers to managing CKD in primary health care (PHC) at both provider and healthcare system levels. Primary care physicians often find it challenging to stay current with evolving CKD guidelines and struggle with limited time, inflexible electronic medical record systems, and scarce resources in their clinics. They desired electronic prompts and lab decision support, concise guidelines, and healthcare financing reform to improve CKD care [7].

Physician knowledge, attitude, and practice regarding CKD have been evaluated in other countries like Cameroon [8] and Jimma, Ethiopia [9], but no studies have been conducted in KSA.

Aim of the research

Given that Internal Medicine (IM), General Practitioners (GPs), and Family Medicine (FM) doctors primarily manage CKD patients, we conducted this cross-sectional study in KSA to assess physicians’ knowledge, attitudes, and the barriers they face in managing CKD. This will improve the management and clinical outcomes of CKD patients.

## Materials and methods

Study design and settings

A cross-sectional study design was conducted using an online Google Form survey in the English language by using a non-probability convenience sampling technique. The survey was distributed to our target physicians through social media platforms, including Twitter, Telegram, and WhatsApp, by sending the survey to physicians' groups or direct messages.

Study population and size

Data were collected from June 2022 to August 2022, and a total of 427 physicians completed the study survey. The study included Saudi and non-Saudi physicians currently working as GPs, FM, or IM physicians, who agreed to participate in the study and excluded physicians in other specialties, medical interns, and healthcare workers other than physicians. The sample size was calculated using OpenEpi software. The minimum required sample size for this study was determined to be 384, assuming that the anticipated frequency is 50%, the precision level is 5%, and the confidence interval is 95%.

Data measurement and collection

The study assessed the physicians' knowledge, attitude, practice, and barriers toward CKD. The questionnaire was adapted from two published studies, and consent was obtained from the authors after contacting them (Table 10) [7,8]. The questionnaire underwent a review by two nephrology experts. It was structured into five sections: socio-demographic data, physicians' knowledge about CKD, physicians' attitudes toward CKD diagnosis and management, physicians' practices regarding CKD diagnosis and management, and physicians' barriers to optimal CKD diagnosis and management.

Statistical analysis

Data were coded and reviewed after extraction. Statistical calculations were done using the computer program SPSS (Statistical Package for the Social Sciences; IBM Corp., Armonk, NY, USA), release 26 for Microsoft Windows. Categorical variables were analyzed using frequencies (number of cases) and valid percentages. The questionnaire included 20 multiple-choice questions (MCQs) related to participants’ knowledge, five MCQs to evaluate participants’ attitudes, and nine MCQs to assess participants’ practices. Each question within the knowledge, attitude, and practice sections was scored dichotomously, awarding one point for each correct response. The total possible scores for knowledge, attitude, and practice were 20, 5, and 9 points, respectively. Physicians' barriers were scored on a Likert scale ranging from 0 to 4. Strongly agree (indicating a low barrier) was assigned a score of 4, while strongly disagree (indicating a high barrier) received a score of 0. The total score for barriers ranged up to 104 points. In addition, the percentage of knowledge, attitude, practice, and barriers was calculated for all participants. Subsequently, the level of knowledge, attitude, practice, and barriers were categorized into three groups: ≤50% represented a low level, 50-70% signified a moderate level, and >70% indicated a high level. Chi-square or Fisher's exact test were used to compare the associated factors with the knowledge, attitude, practice, and barrier levels. P-values less than 0.05 were considered statistically significant.

Ethical considerations

Ethical approval was obtained by the research ethics committee of Taibah University, with the reference number STU-21-023. All participants provided informed consent after receiving a description of the study’s objectives through a questionnaire, and confidentiality was assured.

## Results

General characteristics of study participants

Four hundred twenty-seven physicians participated in the survey, with 60% of them being male and two-thirds aged 30 years or younger. The majority were Saudis, with an equal distribution between those working in governmental hospitals and PHC. Most of them had less than five years of practice. Unfortunately, two-thirds of the physicians stated that they had not attended any clinical courses related to CKD or followed CKD guidelines, despite 79% of them encountering 10 CKD patients on a weekly basis. All data are presented in Table 1.

**Table 1:** Demographic characteristics of physicians FM: Family Medicine; GPs: General Practitioners; IM: Internal Medicine; PHC: Primary Health Care; CKD: Chronic Kidney Disease

Parameters	Category	Count (n=427)	Percentage
Gender	Male	256	60
	Female	171	40
Age	≤ 30 Years	307	71.9
	> 30 Years	120	28.1
Nationality	Saudi	411	96.3
	Non-Saudi	16	3.7
Region	Central	101	23.7
	North	23	5.4
	Southern	45	10.5
	Eastern	85	19.9
	Western	173	40.5
Workplace	PHC	174	40.7
	Governmental hospital	184	43.1
	Private hospital	16	3.7
	University hospital	44	10.3
	Others	9	2.1
Profession	GPs	95	22.2
	Resident	210	49.2
	Registrar	15	3.5
	Senior registrar	55	12.9
	Consultant	52	12.2
Medical specialty	FM	235	55
	GPs	58	13.6
	IM	134	31.4
Years of practice	≤ 5 Years	329	77
	> 5 Years	98	23
CKD patients per week	≤ 10	335	78.5
	> 10	92	21.5
Attending CKD clinical courses	Yes	120	28.1
	No	307	71.9
Following CKD guidelines	Yes	135	31.6
	No	292	68.4

Physicians' knowledge regarding CKD

In Table 2 the questions representing the participants’ knowledge level toward CKD are illustrated. Most physicians demonstrated excellent knowledge about the CKD definition and markers for diagnosing CKD. Additionally, the majority recognized that DM and HTN are risk factors for CKD. However, only half of the respondents were aware that HIV and hepatitis are considered to be CKD risk factors. Meanwhile, two-thirds of physicians knew that CKD is classified into five stages. The majority of participants displayed good knowledge about CKD complications such as uremia, hyperkalemia, HTN, edema, and anemia. Furthermore, most physicians were aware about the various forms of renal replacement therapy (RRT).

**Table 2:** Questions related to the knowledge level of physicians regarding CKD CKD: Chronic Kidney Disease; eGFR: estimated Glomerular Filtration Rate; HIV: Human Immunodeficiency Virus; KDOQI: The Kidney Disease Outcome Quality Initiative; RRT: Renal Replacement Therapy *Correct answers

Parameters	Category			Count (n=427)	Percentage
What is the definition of CKD?	A condition of chronically elevated serum creatinine and urea which is usually reversible with appropriate management.			26	6.1
	Structural or functional kidney damage that can lead to impaired kidney function that persists for ≥3 months with or without alteration of GFR. *			354	82.9
	Irreversible and permanent elevation of serum creatinine.			38	8.9
	Elevation of serum urea.			1	0.2
	Other			2	0.4
	I don't know.			6	1.4
The following are risk factors of CKD?	Diabetes		Yes*	421	98.6
			No	2	0.5
			Don't know	4	0.9
	Drugs		Yes*	406	95.1
			No	15	3.5
			Don't know	6	1.4
	Hypertension		Yes*	420	98.4
			No	3	0.7
			Don't know	4	0.9
	Glomerulonephritis		Yes*	376	88.1
			No	22	5.2
			Don't know	29	6.8
	HIV		Yes*	229	53.6
			No	52	12.2
			Don't know	146	34.2
	Hepatitis		Yes*	167	39.1
			No	110	25.8
			Don't know	150	35.1
The most appropriate marker for kidney function?	Creatinine clearance/eGFR *			374	87.6
	Serum creatinine			48	11.2
	Blood urea nitrogen			3	0.7
	Urine volume			2	0.5
KDOQI Guidelines classify CKD into?	1 Stage			3	0.7
	2 Stages			5	1.2
	3 Stages			10	2.3
	4 Stages			65	15.2
	5 Stages *			267	62.5
	6 Stages			14	3.3
	Don't know			63	14.8
The following are complications of CKD?	Anemia	Yes*		389	91.1
		No		10	2.3
		Don't know		28	6.6
	Hyperkalemia	Yes*		407	95.3
		No		7	1.6
		Don't know		13	3
	Uremia	Yes*		411	96.3
		No		3	0.7
		Don't know		13	3
	Hypertension	Yes*		406	95.1
		No		11	2.6
		Don't know		10	2.3
	Osteodystrophy	Yes*		299	70
		No		16	3.7
		Don't know		112	26.2
	Edema	Yes*		408	95.6
		No		6	1.4
		Don't know		13	3
	Nausea/vomiting	Yes*		339	79.4
		No		32	7.5
		Don't know		56	13.1
	Coma	Yes*		335	78.5
		No		28	6.6
		Don't know		64	15
The following are forms of RRT	Peritoneal dialysis	Yes*		370	86.7
		No		14	3.3
		Don't know		43	10.1
	Kidney transplant	Yes*		329	77
		No		63	14.8
		Don't know		35	8.2
	Hemodialysis	Yes*		408	95.6
		No		2	0.5
		Don't know		17	4

The attitude of physicians toward CKD

The current study revealed a positive attitude among physicians toward CKD, as indicated in Table 3. The majority of participants believed that CKD is a prevalent health issue in KSA (97.7%), with 84.8% considering it a significant problem. Additionally, a large proportion of physicians (88.8%) perceived CKD as a life-threatening disease, and 98.1% recommended routine screening for CKD for patients at high risk of the disease.

**Table 3:** Questions related to the attitude of physicians toward CKD CKD: Chronic Kidney Disease *Correct answers

Parameters	Category	Count (n=427)	Percentage
Do you think CKD is a problem in Saudi Arabia?	Yes*	417	97.7
	No	3	0.7
	Don't know	7	1.6
If yes, it is a	Major problem*	362	84.8
	Minor problem	37	8.7
	I don’t know	28	6.6
What is your attitude towards a patient at risk of developing CKD?	Routine screening for CKD and management of risk factors*	419	98.1
	Watchful waiting/No change in attitude	8	1.9
CKD is life-threatening	Yes*	379	88.8
	No	42	9.8
	Don't know	6	1.4

The practice of physicians toward CKD

One-third (34.2%) of participants screened their patients who are at risk of developing CKD every year, while almost 54% of them screened their patients every six months. The majority of physicians relied on serum creatinine (92.5%) and eGFR (97%) for CKD diagnosis. In contrast, half of the physicians realized that urinalysis (54.8%) and abdominal ultrasound (53.6%) were used to diagnose CKD. Furthermore, most physicians (97.2%) acknowledged that patient education is the appropriate step following a CKD diagnosis.

Nearly 68% of physicians expressed the desire to refer their patients to a nephrologist much earlier than recommended. In contrast, only 16.2% of the participants recognized that stage 4 of CKD is the appropriate time for such a referral to a nephrologist. All data are illustrated in Table 4.

**Table 4:** Questions related to the level of practice of physicians toward CKD CKD: Chronic Kidney Disease; GFR: Glomerular Filtration Rate *Correct answers

Parameters	Category		Count (n=427)	Percentage
How often do you screen patients at risk of developing CKD?	Every month		23	5.4
	Every 6 months		233	54.6
	Every year*		146	34.2
	Never		25	5.9
What means do you use to make your diagnosis of CKD	Serum creatinine	Yes*	395	92.5
		No	32	7.5
	Urinalysis	Yes*	234	54.8
		No	193	45.2
	GFR	Yes*	414	97
		No	13	3
	Abdominal ultrasound	Yes*	229	53.6
		No	198	46.4
When you diagnose CKD in a patient, what are your next steps	Patient education on the disease	Yes*	415	97.2
		No	12	2.8
	Conservative management	Yes*	286	67
		No	141	33
	Referral to a nephrologist	Yes	388	90.9
		No*	39	9.1
The stage should the patient be referred to a Nephrologist	Stage 1		64	15
	Stage 2		50	11.7
	Stage 3		179	41.9
	Stage 4*		69	16.2
	At end stage renal disease		19	4.4
	When symptoms appear		20	4.7
	When a patient needs dialysis		5	1.2
	I don’t know		21	4.9

Barriers among physicians toward CKD 

In Table 5, physicians' responses to the barriers encountered during CKD management are illustrated. In the current study, physicians reported feeling comfortable with various aspects of CKD care, including CKD diagnosis (59%), CKD management (49.9%), CKD patient education (72.8%), avoidance of nephrotoxic medications (80.4%), and managing medication doses (49.6%). They also expressed confidence in managing hypertension (70.7%), anemia (55.3%), hyperkalemia (46.4%), bone disorders (40.1%), and metabolic acidosis (41.2%) in CKD patients.

**Table 5:** Questions related to the level of barriers among physicians toward CKD CKD: Chronic Kidney Disease; NSAIDs: Non-Steroidal Anti-Inflammatory Drugs

Statements	Strongly agree	Agree	Neutral	Disagree	Strongly disagree
I feel comfortable:					
Making the diagnosis of CKD in my patients	103 (24.1%)	149 (34.9%)	122 (28.6%)	44 (10.3%)	9 (2.1%)
Educating my patients about CKD	143 (33.5%)	168 (39.3%)	83 (19.4%)	28 (6.6%)	5 (1.2%)
Managing my patients with CKD	77 (18%)	136 (31.9%)	133 (31.1%)	70 (16.4%)	11 (2.6%)
Managing medication dosing in my patients with CKD	97 (22.7%)	115 (26.9%)	111 (26%)	79 (18.5%)	25 (5.9%)
Avoiding nephrotoxic medications (e.g., NSAIDs) in my patients with CKD	186 (43.6%)	157 (36.8%)	63 (14.8%)	17 (4%)	4 (0.9%)
Managing hypertension in my patients with CKD	137 (32.1%)	165 (38.6%)	84 (19.7%)	34 (8%)	7 (1.6%)
Managing anemia of CKD in my patients	94 (22%)	142 (33.3%)	129 (30.2%)	53 (12.4%)	9 (2.1%)
Managing bone disorders of CKD in my patients	66 (15.5%)	105 (24.6%)	129 (30.2%)	109 (25.5%)	18 (4.2%)
Managing electrolyte disorders (e.g., hyperkalemia) in my patients with CKD	88 (20.6%)	110 (25.8%)	111 (26%)	89 (20.8%)	29 (6.8%)
Managing metabolic acidosis in my patients with CKD	71 (16.6%)	105 (24.6%)	96 (22.5%)	110 (25.8%)	45 (10.5%)
I have available tools (e.g., electronic medical record alerts; checklists; or printed, web-based, or smartphone-based resources) which help me to:					
Diagnose CKD	114 (26.7%)	145 (34%)	95 (22.2%)	49 (11.5%)	24 (5.6%)
Manage CKD	98 (23%)	144 (33.7%)	108 (25.3%)	54 (12.6%)	23 (5.4%)
Manage medication dosing	119 (27.9%)	156 (36.5%)	89 (20.8%)	43 (10.1%)	20 (4.7%)
Avoid prescribing nephrotoxic medications	115 (26.9%)	159 (37.2%)	91 (21.3%)	44 (10.3%)	18 (4.2%)
Manage hypertension in my patients with CKD	107 (25.1%)	166 (38.9%)	97 (22.7%)	42 (9.8%)	15 (3.5%)
Manage anemia of CKD	97 (22.7%)	146 (34.2%)	110 (25.8%)	56 (13.1%)	18 (4.2%)
Manage bone disorders of CKD	79 (18.5%)	129 (30.2%)	123 (28.8%)	68 (15.9%)	28 (6.6%)
Manage hyperkalemia in CKD	98 (23%)	127 (29.7%)	119 (27.9%)	58 (13.6%)	25 (5.9%)
Manage metabolic acidosis in CKD	86 (20.1%)	107 (25.1%)	127 (29.7%)	76 (17.8%)	31 (7.3%)
I have educational tools and resources (e.g., printed and web-based materials/programs, classes, or health educators) available to help my patients understand:					
Their CKD diagnosis	78 (18.3%)	131 (30.7%)	117 (27.4%)	71 (16.6%)	30 (7%)
The potential medication-related risks associated with CKD (e.g., nephrotoxins, medication dosing)					
	72 (16.9%)	121 (28.3%)	132 (30.9%)	64 (15%)	38 (8.9%)
Anemia of CKD	65 (15.2%)	121 (28.3%)	130 (30.4%)	77 (18%)	34 (8%)
Hypertension in CKD	66 (15.5%)	134 (31.4%)	125 (29.3%)	68 (15.9%)	34 (8%)
Bone disorders in CKD patients	58 (13.6%)	115 (26.9%)	127 (29.7%)	93 (21.8%)	34 (8%)
Hyperkalemia in CKD	66 (15.5%)	116 (27.2%)	125 (29.3%)	84 (19.7%)	36 (8.4%)
Metabolic acidosis in CKD	59 (13.8%)	115 (26.9%)	123(28.8%)	91 (21.3%)	39 (9.1%)

Furthermore, two-thirds of the respondents reported having access to the necessary tools for CKD diagnosis (60.7%), CKD management (56.7%), and medication dose management (64.4%), as well as addressing hypertension (64%), anemia (56.9%), bone disorders (48.7%), and hyperkalemia (52.7%) in CKD patients. Approximately 64.1% of physicians reported having sufficient resources to avoid prescribing nephrotoxic medications for their CKD patients. Additionally, 45.2% of the participants reported having effective tools for managing metabolic acidosis among CKD patients.

Moreover, physicians having sufficient educational tools to assist their patients' understanding CKD diagnosis (49%), potential medication-related risks associated with CKD (45.2%), management of HTN (46.9%), hyperkalemia (42.7%), and metabolic acidosis (40.7%), as well as addressing bone disorders (40.5%), and anemia (34.5%) in CKD. 

Level of knowledge, attitude, practice, and barriers among physicians toward CKD

**Figure 1:**
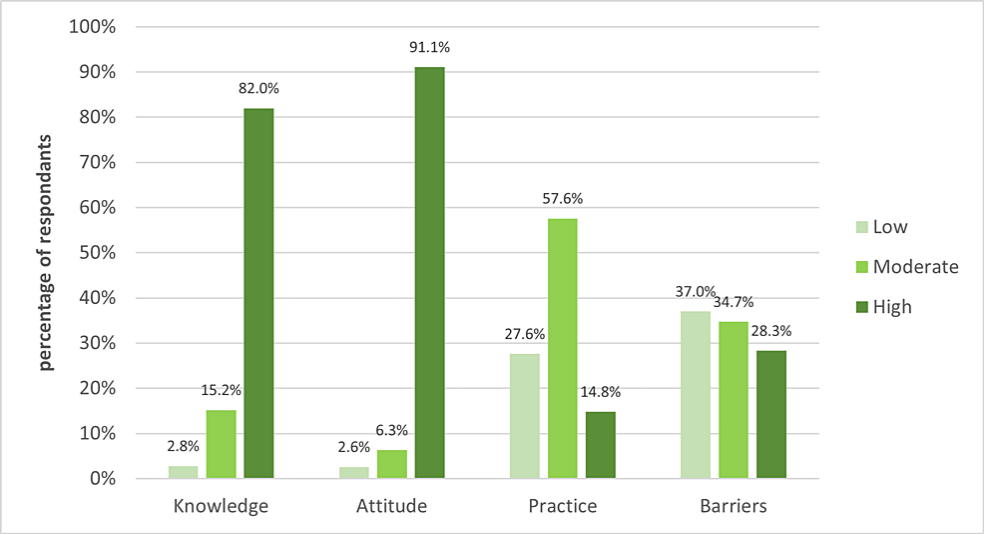
Level of knowledge, attitude, practice, and barrier among physicians toward CKD. CKD: Chronic Kidney Disease

Regarding the assessment of knowledge, attitude, practice, and barrier levels toward CKD among physicians, the current study revealed a high level of knowledge (82%) and positive attitude (91.1%) among participants. Meanwhile, approximately 57.6% of physicians exhibited a moderate level of practice. In addition, around 28.3% of participants reported high barriers to CKD. All data are demonstrated in Figure 1.

Factors related to the level of knowledge among physicians about CKD

The present study investigated the association between various factors and the knowledge level among physicians toward CKD (Table 6). Among different physician professions and specialties, the study revealed that consultants and IM physicians had a statistically significant impact, demonstrating a higher degree of knowledge with a p-value of <0.001 in both groups. Additionally, physicians who adhered to CKD guidelines demonstrated a significantly higher level of knowledge about the disease compared to physicians who did not follow any CKD guidelines (p-value=0.013). In contrast, factors such as gender, age, nationality, workplace, years of practice, number of CKD patients, and attending clinical courses did not have a significant impact on physicians’ knowledge levels.

**Table 6:** Factors related to the level of knowledge among physicians about CKD CKD: Chronic Kidney Disease; PHC: Primary Health Care; FM: Family Medicine; GPs: General Practitioners; IM: Internal Medicine *Fisher test

Factors	Categories	Knowledge level			P-value
		Low	Moderate	High	
Sex	Male	10 (3.9%)	42 (16.4%)	204 (79.7%)	0.170
	Female	2 (1.2%)	23 (13.5%)	146 (85.4%)	
Age	≤30	9 (2.9%)	52 (16.9%)	246 (80.1%)	0.279
	> 30	3 (2.5%)	13 (10.8%)	104 (86.7%)	
Nationality	Saudi	11 (2.7%)	60 (14.6%)	340 (82.7%)	0.090*
	Non-Saudi	1 (6.3%)	5 (31.3%)	10 (62.5%)	
Region	Central	3 (3%)	22 (21.8%)	76 (75.2%)	0.109*
	North	1(4.3%)	6 (26.1%)	16 (69.6%)	
	Southern	1(2.2%)	4 (8.9%)	40 (88.9%)	
	Eastern	1(1.2%)	7 (8.2%)	77 (90.6%)	
	Western	6 (3.5%)	26 (15%)	141 (81.5%)	
Workplace	PHC	3 (1.7%)	28 (16.1%)	143 (82.2%)	0.295*
	Private hospital	1(6.3%)	4 (25.0%)	11(68.8%)	
	Governmental hospital	8 (4.3%)	29 (15.8%)	147 (79.9%)	
	University Hospital	0 (0%)	3 (6.8%)	41 (93.2%)	
	Others	0 (0%)	1 (11.1%)	8 (88.9%)	
Profession	GPs	9 (9.5%)	24 (25.3%)	62 (65.3%)	< 0.001*
	Consultant	1(1.9%)	3 (5.8%)	48 (92.3%)	
	Registrar	0 (0%)	2 (13.3%)	13 (86.7%)	
	Senior registrar	0 (0%)	7 (12.7%)	48 (87.3%)	
	Resident	2 (1%)	29 (13.8%)	179 (85.2%)	
Specialty	FM	5 (2.1%)	40 (17%)	190 (80.9%)	<0.001*
	IM	2 (1.5%)	8 (6%)	124 (92.5%)	
	GPs	5 (8.6%)	17 (29.3%)	36 (62.1%)	
Years of Practice	≤5 years	11(3.3%)	55 (16.7%)	263 (79.9%)	0.116
	>5 years	1 (1%)	10 (10.2%)	87 (88.8%)	
CKD patients per week	≤10 patients	11(3.3%)	54 (16.1%)	270 (80.6%)	0.318
	> 10 patients	1(1%)	11(12%)	80 (87%)	
Attending clinical courses	Yes	3 (2.5%)	17 (14.2%)	100 (83.3%)	0.916
	No	9 (2.9%)	48 (15.6%)	250 (81.4%)	
Follow CKD guidelines	Yes	1 (0.7%)	13 (9.6%)	121 (89.6%)	0.013
	No	11(3.8%)	52 (17.8%)	229 (78.4%)	

Factors related to the level of attitude among physicians 

Regarding attitude levels among participants (Table 7), IM physicians exhibited a significantly more positive attitude toward CKD compared to FM and GPs (p-value=0.035). Additionally, participants who dealt with more than 10 CKD patients per week demonstrated a significantly more positive attitude compared to physicians who managed less than 10 CKD patients per week, with a p-value of 0.038. However, other factors such as gender, age, workplace, profession, years of practice, attending clinical courses, and following CKD guidelines did not have a significant influence on the attitudes of physicians.

**Table 7:** Factors related to the level of attitude among physicians CKD: Chronic Kidney Disease; PHC: Primary Health Care; FM: Family Medicine; GPs: General Practitioners; IM: Internal Medicine *Fisher test

Factors	Categories	Attitude level			P-value
		Low	Moderate	High	
Sex	Male	7 (2.7%)	14 (5.5%)	235 (91.8%)	0.704
	Female	4 (2.3%)	13 (7.6%)	154 (90.1%)	
Age	≤30	7 (2.3%)	19 (6.2%)	281 (91.5%)	0.875
	>30	4 (3.3%)	8 (6.7%)	108 (90%)	
Nationality	Saudi	11(2.7%)	25 (6.1%)	375 (91.2%)	0.532*
	Non-Saudi	0 (0%)	2 (12.5%)	14 (87.5%)	
Region	Central	1 (1%)	9 (8.9%)	91 (90.1%)	0.281*
	North	2 (8.7%)	0 (0%)	21 (91.3%)	
	Southern	1 (2.2%)	5 (11.1%)	39 (86.7%)	
	Eastern	3 (3.5%)	4 (4.7%)	78 (91.8%)	
	Western	4 (2.3%)	9 (5.2%)	160 (92.5%)	
Workplace	PHC	7(4%)	13 (7.5%)	154 (88.5%)	0.154*
	Private hospital	0 (0%)	3 (18.8%)	13 (81.3%)	
	Governmental hospital	3 (1.6%)	10 (5.4%)	171 (92.9%)	
	University hospital	1(2.3%)	0 (0%)	43 (97.7%)	
	Others	0 (0%)	1 (11.1%)	8 (88.9%)	
Profession	GPs	6 (6.3%)	10 (10.5%)	79 (83.2%)	0.053*
	Consultant	0 (0%)	3 (5.8%)	49 (94.2%)	
	Registrar	1 (6.7%)	0 (0%)	14 (93.3%)	
	Senior registrar	0 (0%)	5 (9.1%)	50 (90.9%)	
	Resident	4 (1.9%)	9 (4.3%)	197 (93.8%)	
Specialty	FM	8 (3.4%)	17 (7.2%)	210 (89.4%)	0.035*
	IM	0 (0%)	5 (3.7%)	129 (96.3%)	
	GPs	3 (5.2%)	5 (8.6%)	50 (86.2%)	
Years of Practice	≤5	9 (2.7%)	22 (6.7%)	298 (90.6%)	0.769
	>5	2 (2%)	5 (5.1%)	91 (92.9%)	
CKD patients per week	≤10	11(3.3%)	25 (7.5%)	299 (89.3%)	0.038
	> 10	0 (0%)	2 (2.2%)	90 (97.8%)	
Attending clinical courses	Yes	3 (2.5%)	3 (2.5%)	114 (95%)	0.133
	No	8 (2.6%)	24 (7.8%)	275 (89.6%)	
Follow CKD guidelines	Yes	1 (0.7%)	6 (4.4%)	128 (94.8%)	0.125
	No	10 (3.4%)	21 (7.2%)	261 (89.4%)	

Factors related to practice level among physicians toward CKD

Regarding factors associated with the level of practice, the current study found that IM physicians exhibited statistically significant higher practice levels compared to FM and GPs with p-value=0.018. Furthermore, physicians working in governmental hospitals demonstrated statistically significant higher practice levels with a p-value=0.026 compared to those in other workplaces. Additionally, physicians who are managing ≥10 CKD patients weekly had a significant association with a higher degree of practice than physicians managing less than 10 CKD patients per week (p-value=0.021). Moreover, attending clinical courses and adhering to CKD guidelines had a significant impact on increasing practice levels among physicians compared to those who did not attend any clinical courses on CKD and who did not follow any CKD guidelines, with p-values=0.009 and 0.014, respectively.

On the other hand, factors such as gender, age, years of practice, nationality, and profession did not exhibit a significant influence on the practice level among participants. Data are presented in Table 8.

**Table 8:** Factors related to practice level among physicians toward CKD CKD: Chronic Kidney Disease; PHC: Primary Health Care; FM: Family Medicine; GPs: General Practitioners; IM: Internal Medicine *Fisher test

Factors	Categories	Practice level			P-value
		Low	Moderate	High	
Sex	Male	61(23.8%)	158 (61.7%)	37 (14.5%)	0.073
	Female	57 (33.3%)	88 (51.5%)	26 (15.2%)	
Age	≤30	90 (29.3%)	171 (55.7%)	46 (15%)	0.399
	> 30	28 (23.3%)	75 (62.5%)	17 (14.2%)	
Nationality	Saudi	112 (27.3%)	239 (58.2%)	60 (14.6%)	0.455*
	Non-Saudi	6 (37.5%)	7 (43.8%)	3 (18.8%)	
Region	Central	24 (23.8%)	61 (60.4%)	16 (15.8%)	0.808
	North	6 (26.1%)	13 (56.5%)	4 (17.4%)	
	Southern	13 (28.9%)	24 (53.3%)	8 (17.8%)	
	Eastern	23 (27.1%)	46 (54.1%)	16 (18.8%)	
	Western	52 (30.1%)	102 (59%)	19 (11%)	
Workplace	PHC	56 (32.2%)	99 (56.9%)	19 (10.9%)	0.026*
	Private hospital	9 (56.3%)	6 (37.5%)	1 (6.3%)	
	Governmental hospital	43 (23.4%)	105 (57.1%)	36 (19.6%)	
	University hospital	7 (15.9%)	31 (70.5%)	6 (13.6%)	
	Others	3 (33.3%)	5 (55.6%)	1 (11.1%)	
Profession	GPs	32 (33.7%)	52 (54.7%)	11 (11.6%)	0.218
	Consultant	11(21.2%)	30 (57.7%)	11 (21.2%)	
	Registrar	3 (20%)	12 (80%)	0 (0%)	
	Senior registrar	17 (30.9%)	33 (60%)	5 (9.1%)	
	Resident	55 (26.2%)	119 (56.7%)	36 (17.1%)	
Specialty	FM	71(30.2%)	138 (58.7%)	26 (11.1%)	0.018
	IM	29 (21.6%)	74 (55.2%)	31 (23.1%)	
	GPs	18 (31.0%)	34 (58.6%)	6 (10.3%)	
Years of practice	≤5	99 (30.1%)	182 (55.3%)	48(14.6%)	0.109
	>5	19 (19.4%)	64 (65.3%)	15 (15.3%)	
CKD patients per week	≤10	95 (28.4%)	199 (59.4%)	41 (12.2%)	0.021
	>10	23 (25%)	47 (51.1%)	22 (23.9%)	
Attending clinical courses	Yes	21 (17.5%)	76 (63.3%)	23 (19.2%)	0.009
	No	97 (31.6%)	170 (55.4%)	40 (13%)	
Follow CKD guidelines	Yes	25 (18.5%)	86 (63.7%)	24 (17.8%)	0.014
	No	93 (31.8%)	160 (54.8%)	39 (13.4%)	

Factors associated with the level of barriers among physicians toward CKD

Among different physicians’ specialties and workplaces, GPs and physicians working in PHC centers were found to be significantly associated with higher levels of barriers compared to other groups (p-value=0.016 and <0.001, respectively). In addition, female physicians encountered a higher level of barriers than males with a p-value=0.009. Conversely, IM physicians and physicians treating ≥10 CKD patients weekly faced lower level of barriers compared to other specialties and those treating fewer than 10 CKD patients per week, with a p-value <0.001 in both. Additionally, attending clinical courses and adhering to CKD guidelines demonstrated a statistically significant association with reduced levels of barriers among participants, compared to those who did not attend any clinical courses related to CKD and who did not follow any CKD guidelines (p-value=0.005 and <0.001, respectively) (Table 9). In contrast, factors such as age, years of practice, profession, nationality, and the region in KSA exhibited no significant impact on the level of barriers among the participants.

**Table 9:** Factors associated with the level of barriers among physicians toward CKD CKD: Chronic Kidney Disease; PHC: Primary Health Care; FM: Family Medicine; GPs: General Practitioners; IM: Internal Medicine *Fisher test

Factors	Categories	Barriers level			P-value
		Low	Moderate	High	
Sex	Male	105 (41%)	92 (35.9%)	59 (23%)	0.009
	Female	53 (31%)	56 (32.7%)	62 (36.3%)	
Age	≤30	115 (37.5%)	105 (34.2%)	87 (28.3%)	0.944
	> 30	43 (35.8%)	43 (35.8%)	34 (28.3%)	
Nationality	Saudi	150 (36.5%)	143 (34.8%)	118 (28.7%)	0.502
	Non-Saudi	8 (50%)	5 (31.3%)	3 (18.8%)	
Region	Central	36 (35.6%)	38 (37.6%)	27 (26.7%)	0.469
	North	8 (34.8%)	8 (34.8%)	7 (30.4%)	
	Southern	24 (53.3%)	14 (31.1%)	7 (15.6%)	
	Eastern	31 (36.5%)	29 (34.1%)	25 (29.4%)	
	Western	59 (34.1%)	59 (34.1%)	55 (31.8%)	
Workplace	PHC	38 (21.8%)	74 (42.5%)	62 (35.6%)	<0.001*
	Private hospital	8 (50%)	7 (43.8%)	1 (6.3%)	
	Governmental hospital	87 (47.3%)	54 (29.3%)	43 (23.4%)	
	University hospital	19 (43.2%)	13 (29.5%)	12 (27.3%)	
	Others	6 (66.7%)	0 (0%)	3 (33.3%)	
Profession	GPs	32 (33.7%)	22 (23.2%)	41 (43.2%)	0.016
	Consultant	19 (36.5%)	18 (34.6%)	15 (28.8%)	
	Registrar	4 (26.7%)	5 (33.3%)	6 (40%)	
	Senior registrar	19 (34.5%)	21 (38.2%)	15 (27.3%)	
	Resident	84 (40%)	82 (39%)	44 (21%)	
Specialty	FM	54 (23%)	99 (42.1%)	82 (34.9%)	<0.001
	IM	82 (61.2%)	37 (27.6%)	15 (11.2%)	
	GPs	22 (37.9%)	12 (20.7%)	24 (41.4%)	
Years of Practice	≤5	122 (37.1%)	109 (33.1%)	98 (29.8%)	0.364
	>5	36 (36.7%)	39 (39.8%)	23 (23.5%)	
CKD patients per week	≤10	107 (31.9%)	124 (37%)	104 (31%)	<0.001
	>10	51 (55.4%)	24 (26.1%)	17 (18.5%)	
Attending clinical courses	Yes	59 (49.2%)	33 (27.5%)	28 (23.3%)	0.005
	No	99 (32.2%)	115 (37.5%)	93 (30.3%)	
Follow CKD guidelines	Yes	68 (50.4%)	47 (34.8%)	20 (14.8%)	<0.001
	No	90 (30.8%)	101 (34.6%)	101 (34.6%)	

## Discussion

The current study revealed a notably high level of knowledge among its participants, particularly among consultants, IM physicians, and those who adhered to CKD guidelines. This finding is consistent with a study conducted on healthcare providers, including GPs, resident doctors, specialist doctors, and other health sciences professionals in Jimma, which also demonstrated a good understanding of CKD [9]. However, a cross-sectional study conducted within the West African College of Physicians showed that only one-third of the physicians acquired adequate knowledge of CKD among non-nephrology specialist doctors. Furthermore, there was no significant difference in the level of adequate CKD knowledge between non-nephrology IM and FM physicians [10]. In contrast, a cross-sectional survey involving trainees in IM and FM in the USA highlighted significant knowledge gaps among doctors concerning CKD risk factors, complications, and management [11].

In our study, the majority of respondents demonstrated a strong understanding of the accurate definition of CKD. This contrasts with findings from a study conducted in Cameroon, where only 58.8% of GPs and non-nephrologist specialists correctly recognized the definition of CKD [8]. Additionally, a study focusing on IM residents in the USA revealed that half of them were unaware that kidney injury lasting for three months or more defines CKD [12].

Our physicians exhibited substantial knowledge of the risk factors of CKD, including DM, certain medications, HTN, and glomerulonephritis. However, they demonstrated less recognition of HIV and hepatitis as risk factors for CKD. This finding aligns with a study conducted in Karachi, Pakistan, where GPs were well-informed about DM and HTN as CKD risk factors but showed less awareness of other CKD risk factors [13]. Furthermore, a study carried out in West Africa indicated that all respondents, including FM and non-nephrologist IM physicians, recognized DM and HTN as risk factors for CKD [10].

The most common diagnostic methods employed by our study participants for CKD were serum creatinine and eGFR. A study conducted among non-nephrologist physicians in the United Arab Emirates (UAE) revealed that 77% of physicians utilized eGFR, while 59% of them employed ACR as a screening test for CKD [14]. In Cameroon, 61.4% of the participants opted for eGFR alone or in combination with other tests as a suitable means of diagnosis. Meanwhile, 25.5% of the participants preferred to utilize a combination of serum creatinine, eGFR, urinalysis, and abdominal ultrasound [8].

Our study showed that participants had good knowledge about the complications of CKD. Most participants recognized uremia, hyperkalemia, HTN, edema, and anemia as a complication of CKD which was comparable to other studies [11,13].

Approximately 87.6% of our participants exhibited sound knowledge regarding the most appropriate marker for kidney function, which is creatinine clearance/eGFR. This knowledge stands in contrast to GPs in Pakistan, who preferred serum creatinine as the primary method for assessing kidney function [13].

Approximately two-thirds of the participants in our study did not adhere to CKD guidelines in their clinical practice. A study conducted among Brazilian primary care physicians revealed that half of the physicians were unaware of the national CKD guidelines [15]. Another study conducted in West Africa among FM and non-nephrologist IM physicians found that 37% of them were unaware of any guidelines for the management of CKD, and only 28% were aware of the Kidney Disease Outcome Quality Initiative (KDOQI) guidelines for CKD management [10].

Interestingly, our study found that 91% of physicians displayed a high level of attitude, and 57% exhibited a moderate level of practice towards CKD management. IM physicians and those managing 10 or more CKD patients per week demonstrated a higher level of practice and attitude. Furthermore, physicians working in governmental hospitals, those attending clinical courses, and those who followed CKD guidelines displayed a significant impact on enhancing practice levels among physicians.

The frequency of CKD screening should be personalized, taking into account individual comorbidities, risk profiles, and preferences. Individuals at high risk of developing CKD within five years may benefit from more frequent rescreening, perhaps every one or two years, while those at low risk may require rescreening at more extended intervals [16]. In our study, it was observed that most participants screened for CKD at least every six months, which is considered excessive screening. This approach may have a notable impact on healthcare costs.

The KDIGO guidelines recommend referring patients with CKD to specialized renal care services when their eGFR falls into categories G4-G5, corresponding to eGFR of less than 30 mL/min/1.73m² [1]. However, our study reveals that a majority of respondents tend to refer CKD patients to nephrologists at an earlier stage. While this may have some advantages, it can also lead to an increased burden on the healthcare system. Similarly, a study involving FM and IM physicians demonstrated that only 44.9% of these physicians correctly identified the appropriate eGFR threshold for referral [10]. Another study conducted in the UAE found that 44% of physicians used an eGFR<60 mL/min/1.73m² as an indicator for referring their patients to nephrologists [14].

In our study, approximately 28.3% of participants reported encountering significant CKD-related barriers. GPs working in PHC centers and female physicians were notably associated with a higher level of barriers. Conversely, IM physicians, physicians managing ≥10 CKD patients weekly, those who attended clinical courses, and those who followed CKD guidelines experienced significantly lower barriers. In the current study, most physicians felt comfortable with CKD diagnosis, management, and patient education. Furthermore, the majority of physicians in our study expressed comfort with CKD diagnosis, management, and patient education. They also reported having access to the necessary tools for CKD diagnosis and management. In addition, physicians reported having adequate educational resources to help patients comprehend CKD diagnosis, potential medication-related risks linked to CKD, hypertension management, addressing hyperkalemia, managing metabolic acidosis, bone disorders, and anemia in CKD. Notably, a systematic review of barriers to the detection and management of CKD in primary healthcare settings indicated that common barriers included a lack of time, fear of delivering a CKD diagnosis, and dissatisfaction with CKD guidelines [17]. Another review article emphasized that the absence of training and education in primary healthcare represented a significant barrier to CKD care [18]. Additionally, the identified main barriers encompassed system fragmentation, a lack of updated clinical practice guidelines, and limited training [19].

This study is considered a pioneer one, as it represents the first comprehensive investigation of physicians in KSA regarding their knowledge, attitudes, practices, and barriers related to CKD. It encompasses physicians who directly interact with CKD patients across all regions of KSA. Furthermore, there is a pressing need for additional research that delves deeper into the barriers associated with CKD diagnosis and management. Interventions aimed at addressing these barriers and providing essential support tools have the potential to enhance the effectiveness and capacity of physicians in managing CKD patients. This study also underscores the issue of inappropriate referrals of CKD patients to nephrologists by the majority of the participants. To address this concern, further research is essential to explore the underlying causes behind this practice. Investigating the reasons behind these referrals can lead to more targeted strategies to optimize the healthcare system's utilization and ensure that CKD patients receive timely and appropriate care in the region.

It is important to acknowledge certain limitations in our research. Firstly, the study relied on self-reported data, which may introduce self-reporting bias. Additionally, the convenience sampling method through an online survey may lead to a potential selection bias, as it may not fully represent the broader population of physicians. These limitations might affect the generalizability and reliability of the findings. One other limitation could be that respondents mostly were residents who have not undergone clinical courses in CKD management, this might not be representative of the vast majority of general practitioners in KSA. While we sought to mitigate these limitations, they should be considered when interpreting the results of this study.

## Conclusions

In conclusion, the results of this study conducted in KSA revealed that participants displayed a high level of knowledge and positive attitudes toward CKD. However, a significant proportion of physicians demonstrated a moderate level of practice. Notable areas of concern included poor timing in referrals to nephrologists, with approximately half of the participants screening at-risk patients for CKD every six months rather than annually. While participants encountered low barriers in managing and diagnosing CKD, GPs, FM physicians, and those working in PHC settings faced higher levels of barriers.

## Appendices

**Table 10:** Questionnaire

Section 1: Socio-demographic data						
What is your gender?	Male					
	Female					
What is your age?						
What is your nationality?	Saudi					
	Non-Saudi					
From which region in Saudi Arabia?	Eastern					
	Western					
	Western					
	Central					
	North					
	Southern					
What is your workplace?	1ry health care					
	Governmental hospital					
	Private hospital					
	University hospital					
	Other, please specify:________					
What is your profession?	General practitioners					
	Resident					
	Registrar					
	Senior registrar					
	Consultant					
What is your medical specialty?	Internal medicine					
	Family medicine					
	Other, please specify:________					
How many years have you been in practice?	0 – 5					
	6 -10					
	11- 15					
	More than 15 years					
How many Chronic Kidney Disease (CKD) patients do you see per week?	10 or less					
	11-20					
	21-30					
	31-40					
	More than 40					
Have you attended/participated in any clinical courses regarding CKD during the past year?	Yes					
	No					
Do you follow any chronic kidney disease guidelines?	Yes					
	No					
If yes, which guidelines: __________						
Section 2: Physicians knowledge about CKD						
What is the definition of Chronic Kidney Disease (CKD)	A condition of chronically elevated serum creatinine and urea which is usually reversible with appropriate management					
	Structural or functional kidney damage that can lead to impaired kidney function that persists for 3 months or more with or without alteration of GFR					
	Irreversible and permanent elevation of serum creatinine					
	Elevation of serum urea					
	I don’t know					
	other (specify)					
The following are risk factors of CKD	Diabetes			Yes		
				No		
				don’t know		
	Drugs			Yes		
				No		
				don’t know		
	Hypertension			Yes		
				No		
				don’t know		
	Glomerulonephritis			Yes		
				No		
				don’t know		
	HIV			Yes		
				No		
				don’t know		
	Hepatitis			Yes		
				No		
				don’t know		
The most appropriate marker for Kidney function is	Serum Creatinine					
	Creatinine clearance/estimated Glomerular Filtration Rate					
	Blood Urea Nitrogen					
	Urine volume					
The Kidney Disease Outcome Quality Initiative (KDOQI) Guidelines classify CKD into	1 stage					
	2 stages					
	3 stages					
	4 stages					
	5 stages					
	6 stages					
	I don’t know					
The following are complications of CKD	Anemia			Yes		
				No		
				don’t know		
	Hyperkalemia			Yes		
				No		
				don’t know		
	Uremia			Yes		
				No		
				don’t know		
	Hypertension			Yes		
				No		
				don’t know		
	Osteodystrophy			Yes		
				No		
				don’t know		
	Edema			Yes		
				No		
				don’t know		
	Nausea/vomiting			Yes		
				No		
				don’t know		
	Coma			Yes		
				No		
				don’t know		
The following are forms of Renal Replacement Therapy (RRT)	Peritoneal Dialysis			Yes		
				No		
				I don’t know		
	Kidney Transplant			Yes		
				No		
				I don’t know		
	Hemodialysis			Yes		
				No		
				I don’t know		
Section 2: Physicians knowledge about CKD						
Do you think CKD is a problem in Saudi Arabia?	Yes					
	No					
	I don’t know					
If yes, it is a	Major problem					
	Minor problem					
	I don’t know					
What is your attitude towards a patient at risk of developing CKD?	Routine screening for CKD and management of risk factors.					
	Watchful waiting/No change in attitude					
Do you think that CKD is life-threatening?	Yes					
	No					
	I don’t know					
Section 4: Physicians' practices toward CKD						
How often do you screen patients at risk of developing CKD?	Every month					
	Every 6 months					
	Every year					
	Never					
What means do you use to make your diagnosis of CKD	Serum creatinine			Yes		
				No		
	Urinalysis			Yes		
				No		
	Glomerular Filtration Rate			Yes		
				No		
	Abdominal Ultrasound			Yes		
				No		
If you have other means of diagnosing CKD, please specify						
When you diagnose CKD in a patient, what are your next steps	Patient education on the disease			Yes		
				No		
	Conservative management			Yes		
				No		
	Refer to a Nephrologist			Yes		
				No		
In your opinion, at what stage should the patient be referred to a Nephrologist?	Stage 1					
	Stage 2					
	Stage 3					
	Stage 4					
	At end stage renal disease					
	When symptoms appear					
	When patient needs dialysis					
	I don’t know					
Section 5: Physicians' barriers toward CKD						
Statement	Strongly Agree	Agree	Neutral		Disagree	Strongly Disagree
I feel comfortable:						
Making the diagnosis of chronic kidney disease (CKD) in my patients						
Educating my patients about CKD						
Managing my patients with CKD						
Managing medication dosing in my patients with CKD						
Avoiding nephrotoxic medications (e.g., NSAIDs) in my patients with CKD						
Managing hypertension in my patients with CKD						
Managing anemia of CKD in my patients						
Managing bone disorders of CKD in my patients						
Managing electrolyte disorders (e.g., hyperkalemia) in my patients with CKD						
Managing metabolic acidosis in my patients with CKD						
I have available tools (e.g., electronic medical record alerts; checklists; or printed, web-based, or smartphone-based resources) which help me to:						
Diagnose CKD						
Manage CKD						
Manage medication dosing						
Avoid prescribing nephrotoxic medications						
Manage hypertension in my patients with CKD						
Manage anemia of CKD						
Manage bone disorders of CKD						
Manage hyperkalemia in CKD						
Manage metabolic acidosis in CKD						
I have educational tools and resources (e.g., printed and web-based materials/programs, classes, or health educators) available to help my patients understand:						
Their CKD diagnosis						
The potential medication-related risks associated with CKD (e.g., nephrotoxins, medication dosing)						
Anemia of CKD						
Hypertension in CKD						
Bone disorders in CKD patients						
Hyperkalemia in CKD						
Metabolic acidosis in CKD						

`
